# Altered Lipid Metabolism in Obese Women With Gestational Diabetes and Associations With Offspring Adiposity

**DOI:** 10.1210/clinem/dgac206

**Published:** 2022-03-31

**Authors:** Samuel Furse, Albert Koulman, Susan E Ozanne, Lucilla Poston, Sara L White, Claire L Meek

**Affiliations:** Core Metabolomics and Lipidomics Laboratory, Wellcome Trust-MRC Institute of Metabolic Science, University of Cambridge, Addenbrooke’s Treatment Centre, Keith Day Road Cambridge, CB2 0QQ, UK; Wellcome Trust-MRC Institute of Metabolic Science, University of Cambridge, Addenbrooke’s Treatment Centre, Keith Day Road Cambridge, CB2 0QQ, UK; Core Metabolomics and Lipidomics Laboratory, Wellcome Trust-MRC Institute of Metabolic Science, University of Cambridge, Addenbrooke’s Treatment Centre, Keith Day Road Cambridge, CB2 0QQ, UK; Wellcome Trust-MRC Institute of Metabolic Science, University of Cambridge, Addenbrooke’s Treatment Centre, Keith Day Road Cambridge, CB2 0QQ, UK; Wellcome Trust-MRC Institute of Metabolic Science, University of Cambridge, Addenbrooke’s Treatment Centre, Keith Day Road Cambridge, CB2 0QQ, UK; Department of Women and Children’s Health, School of Lifecourse and Population Sciences, Faculty of Life Sciences and Medicine, King’s College London, London SE1 7EH, UK; Department of Women and Children’s Health, School of Lifecourse and Population Sciences, Faculty of Life Sciences and Medicine, King’s College London, London SE1 7EH, UK; Wellcome Trust-MRC Institute of Metabolic Science, University of Cambridge, Addenbrooke’s Treatment Centre, Keith Day Road Cambridge, CB2 0QQ, UK; Department of Clinical Biochemistry/Wolfson Diabetes & Endocrine Clinic, Cambridge University Hospitals NHS Foundation Trust, Cambridge CB2 0QQUK

**Keywords:** glycemia, lipidomics, triglycerides, de novo lipogenesis, oral glucose tolerance test, pregnancy, gestational diabetes

## Abstract

**Context:**

Gestational diabetes (GDM) affects 20 million women/year worldwide and is associated with childhood obesity. Infants of affected mothers have increased adiposity from birth, which leads to obesity in later life. However, it remains unknown whether the effect of GDM upon neonatal body composition is due to hyperglycemia alone or is mediated by other pathways.

**Objective:**

To investigate plasma lipid profiles in obese women according to GDM diagnosis, infant birthweight percentiles, and adiposity.

**Design:**

Prospective cohort from UPBEAT trial (ISRCTN 89971375).

**Setting:**

Hospital and community.

**Patients:**

867 obese pregnant women recruited in early pregnancy, assessed at 28 weeks for GDM. Offspring anthropometry was assessed at birth.

**Outcome (Prespecified):**

Neonatal birth percentile and abdominal circumference.

**Methods:**

Lipidomic profiling in the fasting plasma oral glucose tolerance test sample using direct infusion mass spectrometry. Analysis included logistic/linear regression, unadjusted and adjusted for maternal age, body mass index, parity, ethnicity, UPBEAT trial arm, and fetal sex. The limit of significance was *P* = 0.05 for offspring anthropometry and *P* = 0.002 for lipidomic data.

**Results:**

GDM in obese women was associated with elevated plasma concentrations of specific diglycerides [DG(32:0)] and triglycerides [TG(48:0), (50:1), (50:2)] containing fatty acids (16:0), (16:1), (18:0), and (18:1), consistent with increased de novo lipogenesis. In the whole cohort, these species were associated with birthweight percentile and neonatal abdominal circumference. Effects upon infant abdominal circumference remained significant after adjustment for maternal glycemia.

**Conclusions:**

Increased de novo lipogenesis-related species in pregnant women with obesity and GDM are associated with measures of offspring adiposity and may be a target for improving lifelong health.

Gestational diabetes mellitus (GDM) is the most common medical complication of pregnancy, affecting 20 million pregnant women worldwide annually (1, 2). Infants born to mothers with GDM are commonly large for gestational age (LGA; >90th percentile for gestational age) with a propensity to higher adiposity at birth, with increased risks of obesity throughout the life course. Early life obesity with accumulation of other cardiovascular risk factors such as glucose intolerance and increased blood pressure in adolescence contributes to a lifetime of multimorbidity, likely to result in a huge international burden of disease.

Despite established associations between obesity, insulin resistance, and adiposity, few studies have explored the potential role of lipid metabolism in the pathophysiology of GDM or in the perinatal or long-term complications for mother and child (1, 3). We previously reported that gestational changes in lipid metabolism are evident in both lean and obese women but are exaggerated in women with obesity (4). We also identified that obese women who develop GDM demonstrate a different metabolomic profile from those who do not, including changes in lipoproteins and their constituents in early and mid-gestation (5, 6). Furthermore, we reported that severity of disease as defined by treatment requirement (insulin vs diet alone) was paralleled by differences in lipid abundance (7).

Other investigators have reported associations between maternal total triglycerides and birthweight in normal-weight and weight-heterogeneous populations (8-10). Beyond known alterations to maternal lipid, inflammatory, and endocrine function (11), obesity is proposed to influence placental lipid biology, including fatty acid (FA) transportation, esterification, oxidation, and placental steatosis, all of which could modulate FA transport to the fetus and influence growth (12). Relationships between maternal obesity, higher fetal triglycerides, and fetal growth have been reported but not consistently (13-15). No previous studies have examined the relationship between specific lipid species, hyperglycemia, and fetal adiposity in the context of maternal obesity.

It has been reported in other types of diabetes that the process of de novo lipogenesis is actively involved in converting excess glucose to new lipid molecules (16). However, the assessment of de novo lipogenesis in pregnancy is challenging. Methods to measure lipogenesis directly using deuterated water are not suitable for use in pregnancy. Therefore, indirect measures of lipogenesis, through identification of lipids including FA(16:0), (16:1), (18:0), and (18:1), are the only safe way to assess de novo lipogenesis activity in pregnancy at scale; have been used in other populations; and show good comparability with measured de novo lipogenesis (17).

The aim of this study was therefore to investigate plasma lipid abundance in obese women with GDM and to assess relationships between lipid species and neonatal adiposity. Specifically, we explored associations of lipid species with GDM, according to GDM severity and tested the hypothesis that there would be a relationship between maternal lipid species related to de novo lipogenesis and measures of neonatal birthweight and neonatal abdominal circumference.

## Materials and Methods

### Study Design

This prospective cohort study was a secondary analysis of the UK Better Eating and Activity Trial (UPBEAT), a randomized controlled trial of a lifestyle intervention in obese pregnant women (ISRCTN 89971375; REC 09/H0802/5). In total, 1555 women aged > 16 years with a body mass index (BMI) ≥ 30 kg/m^2^ with a singleton pregnancy were recruited from 2009 to 2014 and provided written informed consent. Women were randomized between 15^+0^ and 18^+6^ gestational weeks to either a complex behavioral intervention or standard antenatal care as described elsewhere (18). As the intervention did not impact the primary outcomes, GDM or LGA, the trial was treated as a cohort for the purposes of this study. All women with a diagnostic oral glucose tolerance test (OGTT; 24^+2^-30^+0^, mean 27^+5^ gestational weeks) and a research blood sample at the fasting (>10 hours) time point were included. UPBEAT participants were diagnosed with GDM based on the OGTT using the criteria of the International Association of the Diabetes in Pregnancy Study Groups [glucose fasting ≥ 5.1 mmol/L (92 mg/dL); 1-hour ≥ 10.0 mmol/L (180 mg/dL); 2-hour ≥ 8.5 mmol/L (153 mg/dL)]. Women with preeclampsia were excluded a priori. Neonatal birthweight was recorded at birth. LGA was defined as ≥90th percentile using customized birthweight percentiles. Neonatal abdominal circumference (measured at the point of the umbilicus) was measured within 72 hours of delivery by a trained operator (18).

### Lipidomics Analysis and Data Processing

Fasting plasma samples were used for batch lipidomics analysis in randomized order. The lipid, triglyceride, and sterol fractions were isolated together using a high throughput technique developed from existing protocols (19, 20). Samples were infused into an Exactive Orbitrap (Thermo, Hemel Hampstead, UK) using a TriVersa NanoMate (Advion, Ithaca US) for direct infusion mass spectrometry (16). The Exactive Orbitrap acquired data with a scan rate of 1 Hz (resulting in a mass resolution of 100 000 full width at half-maximum at 400 m/z). The instrument was operated in full-scan mode from m/z 150 to 1200 Da. The lipid signals obtained were relative abundance (semi-quantitative), with the signal intensity of each lipid expressed relative to the total lipid signal intensity for each individual, per mille (‰). Raw high-resolution mass-spectrometry data were processed using XCMS (www.bioconductor.org) and Peakpicker v 2.0 (an in-house R script (16)). Lists of known species (by m/z) for both positive ion and negative ion mode were used (~8000 species). Signals that deviated by more than 9 ppm were ignored, as were those with a signal/noise ratio of <3 and those pertaining to <50% of samples. The correlation of signal intensity to concentration of plasma in QCs (0.25, 0.5, 1.0×) was used to identify which lipid signals were linearly proportional to abundance in the sample type and volume used (threshold for acceptance was a correlation of >0.75). Signals were then signal corrected (divided by the sum of signals for that sample) to facilitate comparison of samples. Triglycerides in a plasma sample can undergo fragmentation during analysis and be identified as diglycerides by the mass spectrometer (16). We therefore considered diglycerides in the analysis to most likely represent triglycerides in vivo.

### Statistical Analysis

Maternal characteristics and conventional biochemical analyses were summarized using mean (SD), median (interquartile range), or n (%) as appropriate. We calculated HOMA2b, an index of beta cell function, and HOMA2ir, an index of insulin resistance, using the homeostatic model assessment (available at https://www.dtu.ox.ac.uk/homacalculator/).

An initial investigation of the lipidomics data was performed using sparse partial least squares discriminant analysis and Student’s *t*-test using MetaboAnalyst 5.0 (21). Lipids were regarded as relevant when in the top 10 variables in the loadings of the sparse partial least squares discriminant analysis and had a *P-*value below the Bonferroni-corrected threshold for co-correlated variables [*P* < 0.05/sqrt(n)].

These results were confirmed using regression analyses. To assess the effect of maternal characteristics such as BMI, insulin resistance, and glucose concentrations at OGTT timepoints, we used linear regression with the lipid species as the outcome and the maternal characteristics as explanatory variables. Analyses were presented in unadjusted form. Neonatal outcomes were studied using linear and logistic regression. For assessment of the effect of lipid species on LGA, we used logistic regression with LGA as the outcome and lipid species as explanatory variables. For the assessment of neonatal outcomes measured as continuous variables—specifically, neonatal birthweight percentile and neonatal abdominal circumference—we used linear regression with the continuous variable as an outcome. Where indicated, results are presented in unadjusted form and with minimally adjusted models to assess the effect of a single variable (BMI or fasting glucose) and with a fully adjusted model (maternal age, BMI, parity, ethnicity, fasting glucose, neonatal sex, and trial arm). Neonatal abdominal circumference was also adjusted for estimated gestational age at birth.

Variables that were highly skewed were log-transformed prior to regression analysis. For lipidomic variables we used *P* ≤ 0.002 as the limit of statistical significance based on a modified Bonferroni correction of 0.05/sqrt(n), which makes allowance for multiple testing of 430 highly interdependent lipid variables. For well-recognized neonatal outcomes, we considered *P* ≤ 0.05 to indicate a significant statistical association. Statistical analyses were undertaken using Stata software, version 16.0 (StataCorp LP, College Station, TX, USA). Missing data were not imputed.

## Results

In total, 430 lipid variables were identified including 211 in positive ion mode and 219 in negative ion mode. Women with GDM (n = 241) were of higher age and BMI compared to euglycemic women (n = 626) (Table 1). As anticipated, women with GDM had higher concentrations of insulin and evidence of insulin resistance but clinically comparable results in relation to a standard full lipid profile (Table 1). Women with GDM had offspring with increased birthweight percentile and abdominal circumference (on adjusted analysis).

**Table 1.** Baseline characteristics of euglycemic women and those with gestational diabetes mellitus

	Euglycemic women (n = 626)	Gestational diabetes (n = 241)	*P*-values
Age, years	30.3 (5.7)	31.9 (4.7)	<0.001
BMI, kg/m^2^	34.7 (32.6-38.2)	36.3 (33-39.8)	<0.001
Ethnicity			0.032
White	438 (70.0)	144 (59.8)	
Black	122 (19.5)	59 (24.5)	
Asian	40 (6.4)	22 (9.1)	
Other	26 (4.2)	16 (6.6)	
Nulliparous	291 (46.5)	100 (41.5)	0.19
Trial arm, control	325 (51.9)	125 (51.9)	0.99
Gestational age at OGTT, weeks	27.8 (0.7)	27.7 (1.1)	0.68
Neonatal sex, female	291 (46.5)	118 (50.0)	0.51
Biochemistry			
Fasting glucose	4.5 (4.3-4.8)	5.3 (5.0-5.6)	<0.001
Glucose at 1 hour (OGTT), mmol/L	7.1 (6.0-8.2)	10 (8.6-11.1)	<0.001
Glucose at 2 hours (OGTT), mmol/L	5.6 (4.8-6.2)	6.9 (5.9-7.9)	<0.001
Insulin*, mU/L	17.7 (12.6-30.6)	23.0 (17.2-32.2)	<0.001
C peptide*, nmol//L	1.1 (0.86-1.8)	1.3 (1.1-1.8)	<0.001
Leptin*, ug/L	62.5 (47.4-83.3)	61.8 (46.8-85.8)	0.83
HOMA2b, %	221 (178-314)	197.4 (159-248)	<0.001
HOMA2ir	2.5 (1.8-3.9)	3.3 (2.5-4.5)	<0.001
Total cholesterol*, mmol/L	5.96 (5.31-6.79)	5.95 (5.17-6.71)	0.34
LDL cholesterol*, mmol/L	3.75 (3.09-4.48)	3.66 (2.90-4.39)	0.08
HDL cholesterol*, mmol/L	1.84 (1.58-2.17)	1.76 (1.48-2.09)	0.02
Triglycerides*, mmol/L	1.90 (1.50-2.39)	2.09 (1.64-2.72)	<0.001
Pregnancy outcomes			
Gestational age at birth, weeks	40.3 (39.1-41.1)	39 (38.1-40)	<0.001
Large-for gestational age	43/626 (6.9)	33/241 (13.7)	0.001
Customized birthweight percentile	42.1 (20.3-69.8) n = 626	53.3 (30.0-79.8) n = 241	<0.001
Neonatal abdominal circumference, cm^a^	32.4 (30.9-34.0) n = 295	32.3 (31.1-34.1) n = 122	0.75^a^

Data are presented as mean (SD), median (interquartile range), or n (%) as appropriate. *Analyses performed on the fasting sample at OGTT. Missing data: gestational age at OGTT (n = 2), insulin (n = 3), C-peptide (n = 8), HOMA2b/HOMA2ir (n = 19), total cholesterol and LDL cholesterol (n = 5), HDL cholesterol (n = 4), triglycerides (n = 10), leptin (n = 4), and neonatal abdominal circumference (n = 450).

Abbreviations: BMI, body mass index; HDL, high-density lipoprotein; LDL, low-density lipoprotein; OGTT, oral glucose tolerance test.

^a^Not significant on univariate analysis but significantly different between groups following adjustment for gestational age at birth, ethnicity, and parity (*P* = 0.045, coefficient 0.46; 95% CI 0.01-0.91).

### Plasma Lipid Abundance at 28 Weeks’ Gestation in Obese Women With GDM

**Figure 1.**
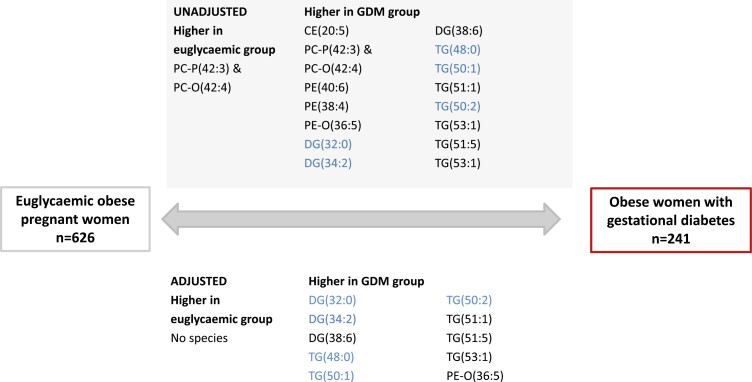
Comparison of lipidomic species abundance in maternal plasma at 28 weeks’ gestation in obese euglycemic women compared to all obese women with gestational diabetes. Triglycerides and diglycerides related to de novo lipogenesis are highlighted in blue.

Lipidomics assessment showed multiple significant differences in lipid species’ abundance between women with GDM compared to euglycemic women (Figure 1) with increases in abundance of specific diglycerides [DG(32:0)] and triglycerides [TG(48:0), (50:1), (50:2)], containing FA(16:0), (16:1), (18:0), and (18:1), which are associated with increased de novo lipogenesis. There was no significant difference in total triglyceride abundance (on lipidomics analysis) between euglycemic women and those with GDM. In the cohort as a whole, increased abundance of lipogenesis-related species was strongly associated with the severity of maternal hyperglycemia at all OGTT timepoints and with maternal insulin resistance but not consistently with maternal BMI (Table 2).

**Table 2.** Associations of lipogenesis-related species, measured using lipidomics, with body mass index and glucose concentrations at intervals post glucose load in the oral glucose tolerance test in obese pregnant women (n = 867)

Lipid species	BMI	Glucose at 0 hours	Glucose at 1 hour	Glucose at 2 hours	Homa2-IR score
DG(32:0)	0.01 (0.00 to 0.03)	0.40 (0.27 to 0.52)^***^	0.11 (0.08 to 0.14)^***^	0.13 (0.08 to 0.17)^***^	0.35 (0.02 to 0.05)^***^
DG(32:1)	0.00 (−0.01 to 0.02)	0.14 (0.00 to 0.27)*	0.03 (−0.01 to 0.07)	0.02 (−0.02 to 0.07)	0.04 (>−0.01 to 0.03)
DG(34:1)	0.01 (0.00 to 0.03)	0.16 (0.03 to 0.29)*	0.07 (0.04 to 0.10)^***^	0.04 (0.00 to 0.09)*	0.02 (>−0.01 to 0.04)
TG(46:0)	0.00 (−0.02 to 0.01)	0.30 (0.17 to 0.43)^***^	0.07 (0.03 to 0.10)^***^	0.07 (0.02 to 0.11)^**^	0.03 (0.01 to 0.05)^**^
TG(46:1)	−0.01 (−0.02 to 0.01)	0.21 (0.09 to 0.34)^***^	0.05 (0.02 to 0.09)^**^	0.04 (−0.01 to 0.08)	0.03 (0.01 to 0.05)^**^
TG(48:0)	0.01 (−0.01 to 0.02)	0.43 (0.30 to 0.55)^***^	0.11 (0.08 to 0.15)^***^	0.12 (0.08 to 0.17)^***^	0.04 (0.02 to 0.06)^***^
TG(48:1)	−0.01 (−0.02 to 0.01)	0.28 (0.15 to 0.41)^***^	0.08 (0.05 to 0.12)^***^	0.07 (0.03 to 0.12)^***^	0.04 (0.02 to 0.06)^***^
TG(50:1)	0.02 (0.01 to 0.04)^**^	0.35 (0.22 to 0.47)^***^	0.11 (0.08 to 0.14)^***^	0.12 (0.07 to 0.16)^***^	0.04 (0.02 to 0.06)^***^
TG(50:2)	0.01 (0.00 to 0.03)*	0.23 (0.11 to 0.36)^***^	0.09 (0.06 to 0.13)^***^	0.10 (0.05 to 0.14)^***^	0.04 (0.02 to 0.06)^***^
Total triglycerides	<0.01 (<0.01 to 0.01)	−0.01 (−0.07 to −0.05)	<0.01 (0.01 to 0.01)	<0.01 (<0.01 to 0.01)	−0.02 (−0.02 to −0.01)^***^

Missing data: glucose at time 1 hour (n = 40); glucose at 2 hours (n = 2), and HOMA2b/HOMA2ir (n = 19).

### Associations With Neonatal Birthweight or Adiposity

To assess whether lipogenesis-related species were associated with fetal size and adiposity, associations between individual species and the development of LGA, neonatal birthweight percentile, and neonatal abdominal circumference were explored in the whole cohort (Table 3). Of the 9 lipogenesis-related species studied [DG(32:0), 32:1), (34:1); TG(46:0), (46:1), (48:0), (48:1), (50:1), (50:2)], significant associations were identified with LGA and birthweight percentile. The associations with birthweight percentile persisted despite adjustment for maternal BMI but were attenuated after adjustment for maternal fasting glucose. Notably, 3 species showed significant associations with neonatal abdominal circumference after adjustment for maternal fasting glucose [DG(32:0); TG(46:0), (46:1)].

**Table 3.** Associations of lipogenesis-related species, measured using lipidomics, in fasting samples of maternal plasma at 28 weeks with neonatal birthweight percentile (n = 865), large-for-gestational age (n = 865), and adiposity (n = 457)

Lipid species	Unadjusted	Adjusted for BMI	Adjusted for fasting glucose	Fully adjusted model
Large for gestational age (n = 865)^a^	Odds ratio (95% CI)	Odds ratio (95% CI)	Odds ratio (95% CI)	Odds ratio (95% CI)
DG(32:0)	1.34 (1.07 to 1.68)*	1.32 (1.05 to 1.66)*	1.20 (0.95 to 1.52)	1.20 (0.94 to 1.52)
DG(32:1)	0.89 (0.65 to 1.22)	0.89 (0.65 to 1.21)	0.86 (0.62 to 1.18)	0.87 (0.63 to 1.20)
DG(34:1)	1.06 (0.84 to 1.34)	1.05 (0.83 to 1.32)	1.02 (0.79 to 1.31)	1.01 (0.77 to 1.32)
TG(46:0)	1.12 (0.90 to 1.39)	1.12 (0.90 to 1.39)	1.03 (0.82 to 1.30)	1.04 (0.83 to 1.32)
TG(46:1)	1.06 (0.83 to 1.35)	1.07 (0.84 to 1.36)	1.00 (0.79 to 1.27)	1.01 (0.79 to 1.29)
TG(48:0)	1.26 (1.03 to 1.54)*	1.25 (1.02 to 1.54)*	1.13 (0.91 to 1.40)	1.14 (0.92 to 1.42)
TG(48:1)	1.15 (0.91 to 1.46)	1.16 (0.92 to 1.47)	1.06 (0.84 to 1.35)	1.07 (0.84 to 1.38)
TG(50:1)	1.37 (1.08 to 1.74)^**^	1.34 (1.05 to 1.69)*	1.24 (0.97 to 1.58)	1.23 (0.95 to 1.58)
TG(50:2)	1.27 (0.99 to 1.62)	1.24 (0.97 to 1.60)	1.18 (0.92 to 1.52)	1.16 (0.89 to 1.53)
Total triglycerides	1.23 (1.02 to 1.48)*	1.23 (0.81 to 1.85)	1.27 (0.84 to 1.92)	1.26 (0.83 to 1.93)
Neonatal birthweight percentile^b^ (n = 865)	Coef (95% CI)	Coef (95% CI)	Coef (95% CI)	Coef (95% CI)
DG(32:0)	2.84 (0.87 to 4.81)^**^	2.67 (0.70 to 4.64)^**^	1.42 (−0.55 to 3.38)	1.29 (−0.70 to 3.28)
DG(32:1)	0.46 (−1.64 to 2.56)	0.43 (−1.66 to 2.53)	−0.06 (−2.10 to 1.98)	−0.19 (−2.25 to 1.87)
DG(34:1)	1.65 (−0.34 to 3.63)	1.52 (−0.46 to 3.50)	1.06 (−0.88 to 2.99)	0.91 (−1.04 to 2.87)
TG(46:0)	2.71 (0.74 to 4.68)^**^	2.75 (0.78 to 4.71)^**^	1.61 (−0.33 to 3.56)	1.61 (−0.35 to 3.58)
TG(46:1)	1.98 (0.00 to 3.95)*	2.09 (0.13 to 4.06)*	1.18 (−0.75 to 3.11)	1.18 (−0.81 to 3.16)
TG(48:0)	2.93 (0.96 to 4.90)^**^	2.86 (0.89 to 4.82)^**^	1.38 (−0.59 to 3.35)	1.33 (−0.66 to 3.31)
TG(48:1)	1.68 (−0.29 to 3.66)	1.77 (−0.20 to 3.74)	0.64 (−1.30 to 2.59)	0.62 (−1.40 to 2.64)
TG(50:1)	1.76 (−0.21 to 3.74)	1.51 (−0.47 to 3.49)	0.47 (−1.49 to 2.43)	0.31 (−1.71 to 2.32)
TG(50:2)	0.73 (−1.24 to 2.71)	0.55 (−1.42 to 2.53)	−0.15 (−2.09 to 1.79)	−0.40 (−2.45 to 1.64)
Total triglycerides	−0.81 (−2.65 to 1.02)	1.19 (−2.63 to 5.02)	1.67 (−2.06 to 5.40)	1.56 (−2.20 to 5.31)
Neonatal abdominal circumference (n = 457)^b^	Coef (95% CI)	Coef (95% CI)	Coef (95% CI)	Coef (95% CI)
DG(32:0)	0.28 (0.07 to 0.49)^**^	0.25 (0.04 to 0.46)*	0.27 (0.06 to 0.49)*	0.30 (0.09 to 0.51)^**^
DG(32:1)	0.11 (−0.10 to 0.32)	0.11 (−0.09 to 0.32)	0.10 (−0.10 to 0.31)	0.12 (−0.09 to 0.32)
DG(34:1)	0.20 (−0.01 to 0.40)	0.19 (−0.02 to 0.39)	0.19 (−0.01 to 0.39)	0.16 (−0.03 to 0.36)
TG(46:0)	0.23 (0.00 to 0.46)	0.24 (0.01 to 0.47)*	0.22 (−0.01 to 0.46)	0.26 (0.04 to 0.50)*
TG(46:1)	0.24 (0.04 to 0.44)*	0.27 (0.07 to 0.47)^**^	0.24 (0.04 to 0.44)*	0.26 (0.07 to 0.46)^**^
TG(48:0)	0.19 (−0.04 to 0.41)	0.17 (−0.05 to 0.40)	0.18 (−0.05 to 0.41)	0.24 (0.01 to 0.46)*
TG(48:1)	0.19 (−0.02 to 0.40)	0.20 (−0.01 to 0.41)	0.18 (−0.03 to 0.39)	0.30 (0.09 to 0.51)^**^
TG(50:1)	0.23 (0.02 to 0.44)*	0.19 (−0.02 to 0.40)	0.22 (0.01 to 0.43)*	0.12 (−0.09 to 0.32)
TG(50:2)	0.26 (0.04 to 0.47)*	0.23 (0.02 to 0.45)*	0.25 (0.04 to 0.47)*	0.16 (−0.03 to 0.36)
Total triglycerides	−0.03 (−0.25 to 0.18)	−0.14 (−0.51 to 0.22)	−0.10 (−0.47 to 0.28)	−0.06 (−0.43 to 0.31)

The fully adjusted model has been adjusted for maternal age, BMI, parity, ethnicity, fasting glucose, neonatal sex and trial arm (and gestational age at delivery for neonatal abdominal circumference). Missing data: large for gestational age/ birthweight percentile (n = 2), gestational age at OGTT (n = 2), neonatal abdominal circumference (n = 410). *P  ≤ 0.05; **P ≤ 0.01; ***P ≤ 0.001.

Abbreviations: BMI, body mass index; DG, diglyceride; TG, triglyceride.

^a^Results are odds ratios (95% CI).

^b^Results are regression coefficients (95% CI) for 1 SD increase in each lipid species.

## Discussion

### Summary of Findings

We have shown that obese women with GDM have an increased plasma abundance of lipid species associated with de novo lipogenesis (22), including specific diglycerides [DG(32:0)] and triglycerides [TG(48:0), (50:1), (50:2)] compared to euglycemic obese pregnant women. The plasma abundance of these lipid species in the whole cohort was strongly related to the severity of maternal hyperglycemia and insulin resistance but was not associated with maternal BMI. Plasma abundance of lipogenesis-associated species was associated with birthweight percentile and neonatal abdominal circumference in the whole cohort. Notably, associations with LGA and birthweight percentile did not persist after adjustment for glucose, whereas the relationship between lipogenesis-associated species and neonatal abdominal circumference was maintained. This suggests that de novo lipogenesis is a key feature of GDM pathophysiology in obese pregnancy and may contribute to the relationship between GDM and measures of offspring adiposity independent of hyperglycemia.

### Strengths and Limitations

A key strength of this analysis is the inclusion of a large number of prospectively collected samples from a well-characterized multiethnic cohort, representative of typical clinical populations. Samples were taken at the time of GDM diagnosis, which removes the possibility of bias from treatment or behavioral change associated with GDM management. Not all women recruited to UPBEAT provided a research sample at the OGTT, and several samples had inadequate volume for analysis. Lipidomics analysis in maternal plasma provides an indication of lipid supply and metabolism, but the lack of direct assessment of de novo lipogenesis could be considered a limitation, although this would be unfeasible in such a large pregnant cohort. As women were participants in a randomized controlled trial, we included adjustments for trial arm, although the intervention did not influence the development of hyperglycemia (18). The lipidomic analysis was performed on a fasting sample, and postload plasma samples may show different lipid changes, but samples for 1- or 2-hour timepoints were not available. Other work suggests that postprandial lipids may be more strongly predictive of neonatal fat mass than fasting values (23, 24). Ethnicity is known to affect lipid homeostasis (25), but subanalysis by ethnicity was also precluded by small numbers. This study benefits from neonatal outcomes related to size and adiposity at birth, but these measures are also likely to be influenced by other factors, including maternal glycemia at 28 to 36 weeks and maternal treatment for GDM. We consider abdominal circumference to be an indirect measure of infant adiposity (26).

### Meaning of the Study Results

#### Lipid profiles in GDM

Previous work by Barbour et al identified strong associations between maternal serum triglycerides in early pregnancy and newborn adiposity in obese women (23), but lipid homeostasis in GDM has been studied in less detail. Conventional clinical laboratory analyses revealed no important differences in lipid profiles in women with GDM compared to euglycemic women (Table 1). In contrast, lipidomics revealed multiple lipid species associated with de novo lipogenesis that were more abundant in women with GDM (Fig. 1). Serum lipid species arise predominantly from a combination of maternal diet, maternal lipolysis, and de novo lipogenesis. In health, de novo lipogenesis is an important and highly regulated pathway that enables glucose to be converted to FAs [typically FA(16:0) and FA(18:0)] in adipose or hepatic tissue. The resulting FAs can be stored as TGs, providing a fuel source via beta oxidation when glucose is less abundant, for example, in the fasting state. Glucose acutely increases de novo lipogenesis by enhancing glycolysis, but in the longer term, chronic hyperglycemia and hyperinsulinemia both influence transcription of key pathway enzymes that increase de novo lipogenesis.

Excess activity of the de novo lipogenesis pathway has been associated with obesity, insulin resistance, type 2 diabetes, and nonalcohol-related fatty liver disease in nonpregnant adults (22, 27-30) but has not previously been reported in serum from women with GDM. Lipid physiology in pregnancy is complex, as both fetus and placenta play an active role, with enhanced de novo lipogenesis occurring in early pregnancy (31-33).While de novo lipogenesis is generally considered to be a response to excess dietary carbohydrate (34), the propensity to increased de novo lipogenesis activity observed in obese pregnant women may have had a causal role in the development of their obesity and hence GDM. Genetic studies of GDM risk have consistently identified associations with genes related to pancreatic beta cell function and insulin synthesis (35), but a recent report identified a liver lipid-related gene cluster related to GDM (36).

The tissue of origin for the increased synthesis of new FAs identified is unknown. In nonpregnant adults, most de novo lipogenesis occurs in liver or adipose, but in pregnancy, fetal or placental tissue may also contribute. De novo lipogenesis has been reported in fetal tissue from 16 weeks’ gestation, activated by insulin (37). The placenta also plays an active role in lipid metabolism (38, 39). One report has suggested that placental FA synthase and the sex hormone response element binding protein, key components of the de novo lipogenesis pathway, are differentially regulated in women with GDM compared to euglycemic women (40).

#### Lipid profiles and neonatal outcomes

Neonatal abdominal circumference is associated with superficial abdominal adipose, deep subcutaneous adipose, and intraorgan/visceral adipose depots (26). Our findings corroborate conventional wisdom that LGA, birthweight percentile, and neonatal abdominal circumference are associated with maternal glycemia, likely through fetal hyperinsulinemia. However, the association between lipogenesis-related species and neonatal adiposity as assessed indirectly by neonatal abdominal circumference is novel and suggests that specific lipid species may influence body composition, independent of maternal hyperglycemia. Further work is needed to clarify whether specific lipid species are associated with specific intrahepatic lipid accumulation or visceral adiposity, a process that could have lifelong metabolic effects in the offspring.

The associations between de novo lipogenesis and body composition require validation in other cohorts. Maternal obesity, GDM, insulin resistance, and a high-fat diet have not only been associated with childhood obesity but also in neonates and in experimental animals with liver fat accumulation in adult offspring [reviewed in (27)]. In rodents, this shares similarities with pediatric nonalcohol-related fatty liver disease (41), a condition known to be associated with de novo lipogenesis (27). Having shown a strong relationship between maternal BMI and adiposity in the 3-year old UPBEAT offspring (42), we are now positioned to address a potential mediating role for maternal lipogenesis species and liver fat accumulation in a planned study of 9- to 11-year-old UPBEAT children.

## Conclusion

De novo lipogenesis-related lipid species were more abundant in obese women with GDM and may contribute to the relationship with offspring adiposity, independently of maternal glucose. Targeting the de novo lipogenesis pathway in pregnancy may improve offspring cardiometabolic health and reduce later life multimorbidity.

## Data Availability

Data are available upon request, subject to approval from the UPBEAT consortium.

## Abbreviations

CE: cholesteryl ester
DG: diglyceride
GDM: gestational diabetes
OGTT: oral glucose tolerance test
PC: phosphatidylcholine
PE: phosphatidylethanolamine
PG: phosphatidylglycerol
PI: phosphatidylinositol
PS: phosphatidylserine
SM: sphingomyelin
TG: triglyceride

## Prior Publication

An earlier form of this work in abstract form was presented as a poster at the Diabetes UK conference, 2021.

## Clinical Trial Information

UPBEAT trial: ISRCTN 89971375.
